# Bile acid TUDCA improves insulin clearance by increasing the expression of insulin-degrading enzyme in the liver of obese mice

**DOI:** 10.1038/s41598-017-13974-0

**Published:** 2017-11-01

**Authors:** Jean Franciesco Vettorazzi, Mirian Ayumi Kurauti, Gabriela Moreira Soares, Patricia Cristine Borck, Sandra Mara Ferreira, Renato Chaves Souto Branco, Luciana de Souza Lima Michelone, Antonio Carlos Boschero, Jose Maria Costa Junior, Everardo Magalhães Carneiro

**Affiliations:** Department of Structural and Functional Biology, Institute of Biology, University of Campinas (UNICAMP), 13083-970 Campinas, SP Brazil

## Abstract

Disruption of insulin secretion and clearance both contribute to obesity-induced hyperinsulinemia, though reduced insulin clearance seems to be the main factor. The liver is the major site for insulin degradation, a process mainly coordinated by the insulin-degrading enzyme (IDE). The beneficial effects of taurine conjugated bile acid (TUDCA) on insulin secretion as well as insulin sensitivity have been recently described. However, the possible role of TUDCA in insulin clearance had not yet been explored. Here, we demonstrated that 15 days treatment with TUDCA reestablished plasma insulin to physiological concentrations in high fat diet (HFD) mice, a phenomenon associated with increased insulin clearance and liver IDE expression. TUDCA also increased IDE expression in human hepatic cell line HepG2. This effect was not observed in the presence of an inhibitor of the hepatic membrane bile acid receptor, S1PR2, nor when its downstream proteins were inhibited, including IR, PI3K and Akt. These results indicate that treatment with TUDCA may be helpful to counteract obesity-induced hyperinsulinemia through increasing insulin clearance, likely through enhanced liver IDE expression in a mechanism dependent on S1PR2-Insulin pathway activation.

## Introduction

Obesity is the primary cause of hyperinsulinemia, which increases the risk for cancer and cardiovascular diseases^1,2^ and potentiates insulin resistance that may trigger type 2 diabetes (T2D)^3^. High levels of plasma insulin concentration can be attributed to increased insulin secretion and/or decreased insulin clearance^4,5^; however, there is evidence that reduced insulin clearance is likely the primary factor in obesity-induced hyperinsulinemia^6^.

Insulin clearance occurs mainly in the liver by the action of insulin-degrading enzyme (IDE), which degrades approximately 50% of insulin in its first passage through the hepatic portal system^7,8^. IDE is a zinc metalloproteinase which degrades not only insulin but also other amiloidogenic peptides such as glucagon^9^, amylin^10^ and amyloid β^11^. Thus, malfunction of this enzyme is associated with T2D and Alzheimer’s Diseases (AD)^12,13^.

While there is a consensus that increasing IDE function in AD patients could be useful to treat this pathology, this same therapeutic approach is uncertain for patients with T2D. Improvement of insulin signaling and glucose tolerance was observed in mice treated with the IDE inhibitor 6bK^14^. However, treatment with BDM44768, another IDE inhibitor, impaired glucose tolerance, despite increasing insulin signaling^15^. In addition, IDE-deficient mice display chronic hyperinsulinemia that induces, over time, glucose intolerance as well as insulin resistance^16^, suggesting that the hyperinsulinemic state, due to IDE deficiency, could be a trigger for the development of T2D. In the same way, Goto-Kakizaki rats (a non-obese T2D animal model), which have a defect at the IDE gene, as well as some type 2 diabetic patients, exhibit reduced insulin clearance and augmented plasma insulin concentrations prior to the onset of T2D^17,18^. Therefore, we believe that therapeutic strategies focusing on increased IDE expression and insulin clearance could be helpful in the prevention and/or treatment of T2D, especially when hyperinsulinemia precedes the development of this pathology.

In this sense, insulin sensitizer agents such as physical exercise, bariatric surgery and pioglitazone treatment have been found to reduce plasma insulin concentrations in obese rodents, through increased insulin clearance and improved glucose homeostasis^19–21^. However, exercise has a low adherence rate^22^, bariatric surgery is an invasive procedure^23^, and pioglitazone treatment has significant side effects^24^. Thus, the use of endogenous molecules that increase insulin clearance, without the side effects or adherence concerns, could be a potential treatment for hyperinsulinemia.

In this context, the taurine conjugated bile acid tauroursodeoxycholic (TUDCA) has emerged as a possible candidate due to its beneficial effect upon glucose homeostasis^25–27^. In the liver, TUDCA improves insulin sensitivity by reducing endoplasmic reticulum (ER) stress^28,29^. Also, TUDCA activates insulin signaling in the liver, by the interaction with the sphingosine-1-phosphate receptor 2 (S1PR2), resulting in PI3K/Akt pathway activation^30^. However, the effect of TUDCA upon insulin clearance, as well as hepatic IDE expression remains unclear.

Here, using high fat diet (HFD) mice as an experimental model of hyperinsulinemia, we demonstrated that treatment with TUDCA normalizes their plasma insulin concentrations by increasing insulin clearance. This effect is probably due to increased IDE expression in the liver. *In vitro* experiments, using hepatic human cell line HepG2, demonstrated that TUDCA also increases IDE expression, by a mechanism dependent on the interaction of TUDCA with the S1PR2 receptor, via the insulin signaling pathway. These findings suggest treatment with TUDCA as a promising therapeutic intervention for the control of hyperinsulinemia in obese pre-diabetic individuals.

## Results

### TUDCA reduced body weight, fat pad weight and blood glucose in HFD mice

As expected, body weight was significantly increased in HFD, compared with CON mice (Table 1). This effect was accompanied by higher perigonadal and retroperitoneal fat pad weight, as well as higher fed/fasted blood glucose concentrations. TUDCA treatment reduced body and fat pad weight in the HFD + TUDCA mice (Table 1) and also returned fed/fasted blood glucose concentrations to levels similar to the CON mice (Table 1). However, TUDCA treatment did not alter all these parameters in CON + TUDCA mice, corroborating previous studies^29,31^.

**Table 1:** Final characterization of CON, CON + TUDCA, HFD and HFD + TUDCA mice.

	CON	CON + TUDCA	HFD	HFD + TUDCA
Body Weight (g)	30.33 ± 1.21	29.68 ± 1.48	41.22 ± 1.18^*^	33.88 ± 1.27*^#^
Perigonadal fat pad weight (g)	0.314 ± 0.03	0.276 ± 0.02	1.309 ± 0.10^*^	0.8746 ± 0.06^#^
Retroperitoneal fat pad weight (g)	0.154 ± 0.01	0.133 ± 0.01	0.630 ± 0.06^*^	0.336 ± 0.03*^#^
Fasted Glycemia (mg/dL)	90 ± 4.32	91.5 ± 10.0	111 ± 5.42^*^	89.38 ± 5.56
Fed Glycemia (mg/dL)	137 ± 7.78	121 ± 6.02	161 ± 2.00^*^	138 ± 6.48

(*)Indicates statistic differences compared to CON, and ^(#)^Indicates differences between HFD and HFD + TUDCA (One-way ANOVA followed by Newmans-Keuls posttest, P < 0.05). Data are mean ± SEM (n = 4–8).

### TUDCA improved glucose tolerance and insulin sensitivity in HFD mice

**Figure 1:**
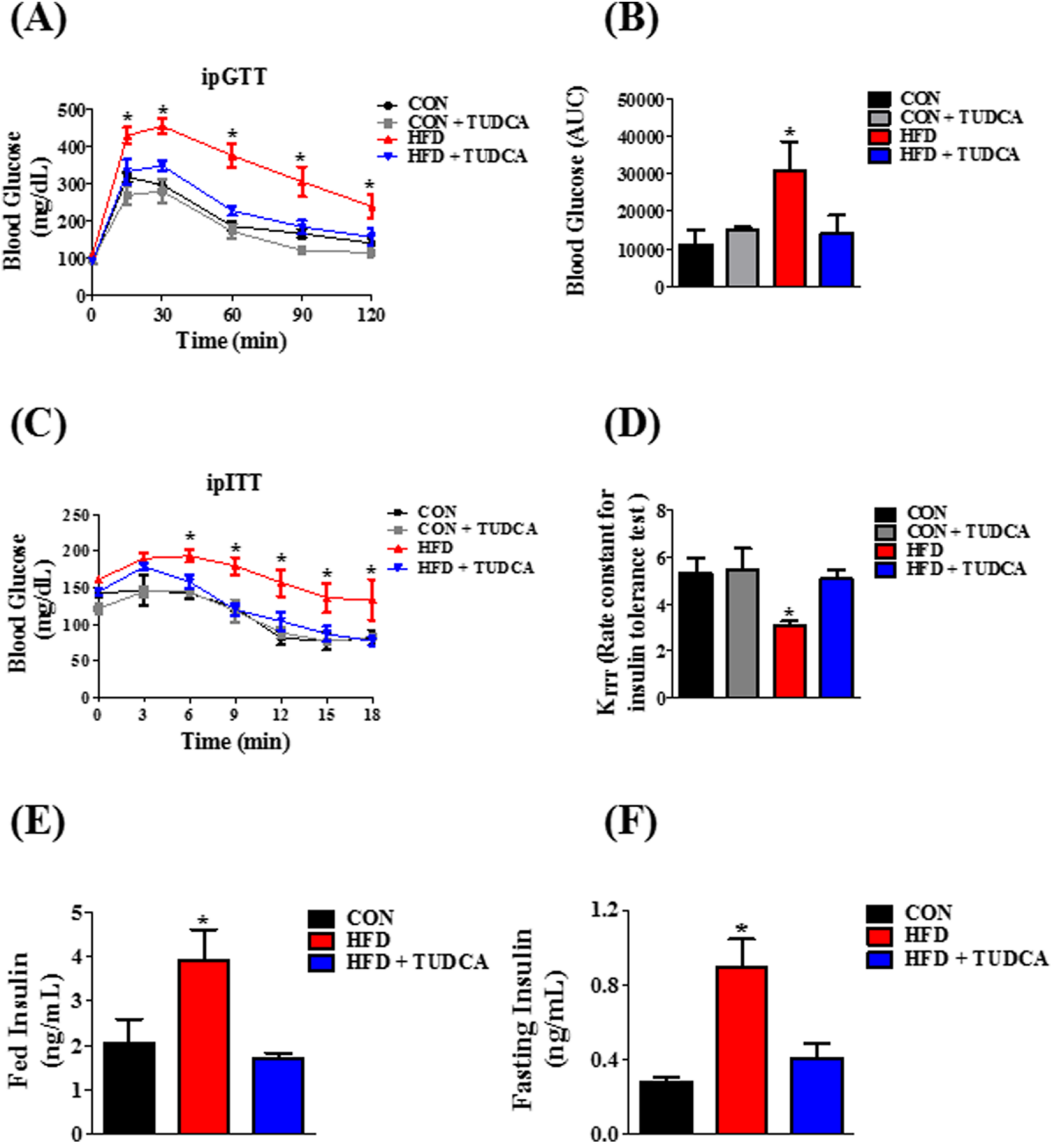
TUDCA treatment improves glucose tolerance, insulin sensitivity and insulinemia in HFD mice. Blood glucose during ipGTT (**A**) and ipITT (**C**). Area under the curve (AUC) of total blood glucose concentration during ipGTT (**B**) and glucose disappearance rate during ipITT (KITT) (**D**). Plasma insulin in fed (**E**) and fasting (**F**) state. Mice were fed a control diet (CON and CON + TUDCA) or high fat diet (HFD and HFD + TUDCA) for 12 weeks, and received or not i.p. 300 mg/kg TUDCA during 15 days, as indicated. Data are mean ± SEM (n = 4–8). *P ≤ 0.05 *vs* CON.

To investigate the effects of TUDCA on glucose homeostasis, we performed intraperitoneal glucose and insulin tolerance tests (ipGTT and ipITT). After the glucose load, during ipGTT, all groups had a maximal glucose peak at 15–30 min (Fig. 1A). However, HFD mice presented higher blood glucose concentrations indicating an impairment of glucose tolerance, as judged by the higher AUC of blood glucose, compared with the other groups (Fig. 1B). Interestingly, HFD + TUDCA mice presented an improved glucose tolerance (Fig. 1A), as observed by the lower AUC of blood glucose, during ipGTT (Fig. 1B). During the ipITT, HFD mice displayed higher blood glucose, compared with CON mice (Fig. 1C), suggesting impaired insulin sensitivity in these HFD mice, as judged by the glucose disappearance rate (KITT) (Fig. 1D). The treatment with TUDCA restored insulin sensitivity in HFD + TUDCA mice (Fig. 1D), increasing the KITT values (Fig. 1D). Finally, we assessed plasma insulin levels in the fed and fasted state and we observed that HFD increased plasma insulin concentrations in both states (Fig. 1E and F) and the treatment with TUDCA restored this parameter in HFD + TUDCA to levels similar to those found in CON mice (Fig. 1E and F).

### TUDCA increased insulin clearance and IDE expression, but not IDE activity, in the liver of HFD mice

**Figure 2:**
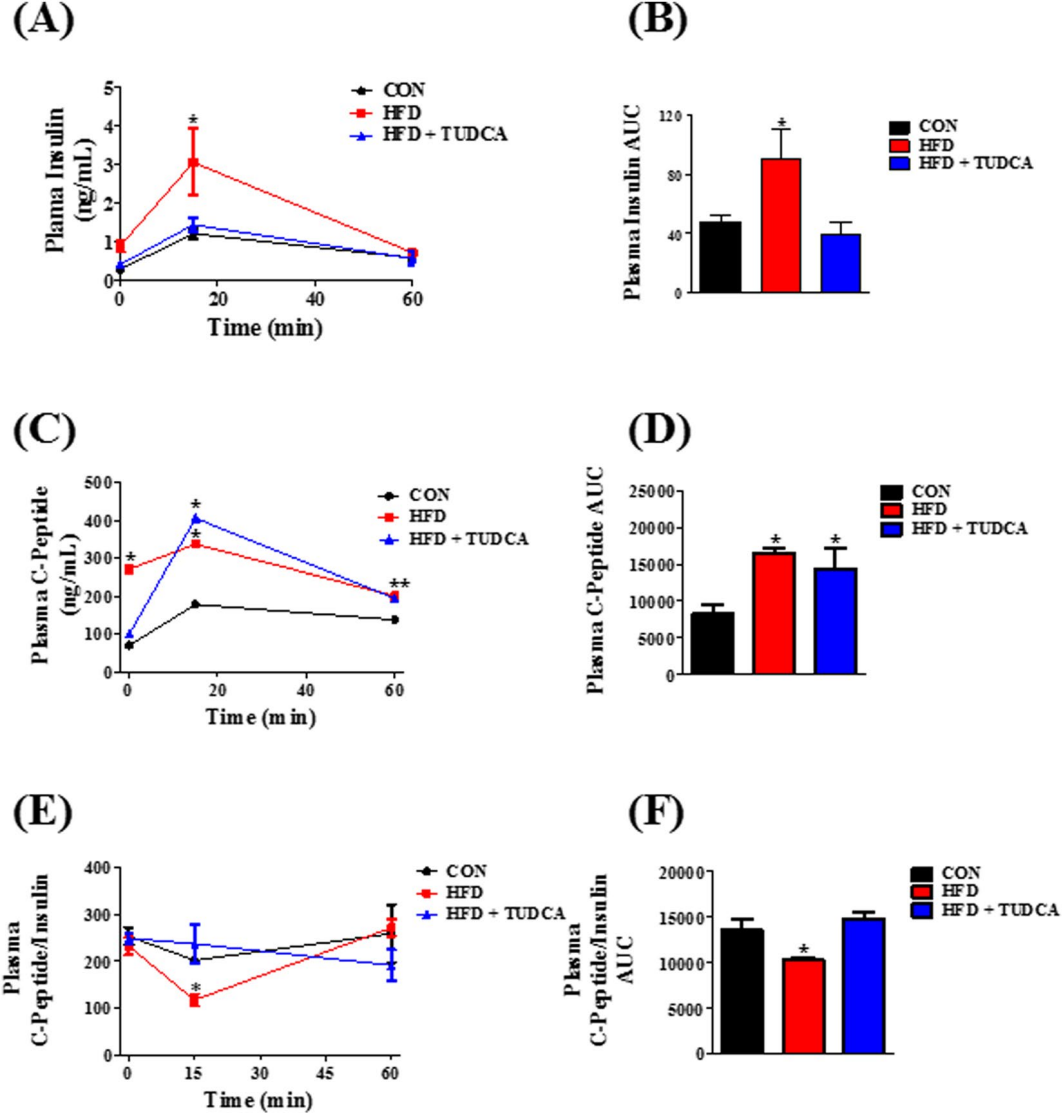
TUDCA treatment increases insulin clearance in HFD mice. Plasma levels of insulin (**A**), C-peptide (**C**) and the C-peptide/Insulin ratio (**E**). AUC of plasma insulin concentration (**B**), C-peptide (**D**) and C-peptide/Insulin ratio (**F**). Mice were fed a control diet (CON) or high fat diet (HFD and HFD + TUDCA) for 12 weeks, and received or not i.p. 300 mg/kg TUDCA during 15 days, as indicated. Data are mean ± SEM (n = 4–8). *P ≤ 0.05 *vs* CON.

**Figure 3:**
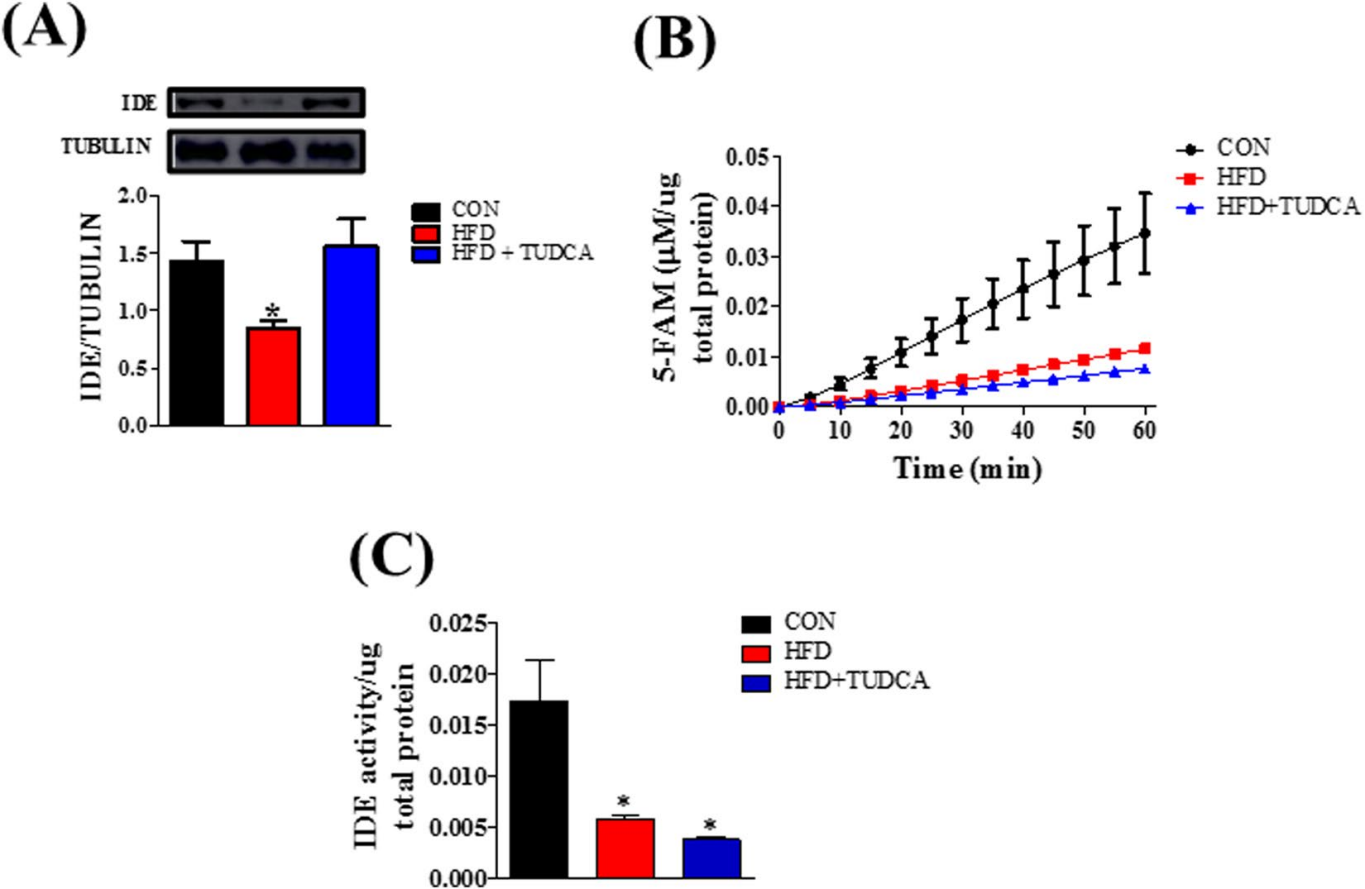
TUDCA treatment increases IDE expression, but not activity in HFD mice. Protein expression of IDE in the liver and its representative immunoblotting images (**A**). Kinetic of IDE activity assay (**B**) and total IDE activity (**C**) in the liver of mice. Fluorescent intensity at Ex/Em = 490/520 nm was recorded, every 5 min, during 60 min. 5-FAM concentration was calculated using a standard curve and normalized per μg of total protein. Mice were fed a control diet (CON) or high fat diet (HFD and HFD + TUDCA) for 12 weeks, and received or not i.p. 300 mg/kg TUDCA during 15 days, as indicated. Data are mean ± SEM (n = 4–8). Data are mean ± SEM (n = 4–8). *P ≤ 0.05 *vs* CON.

Plasma insulin concentration is controlled by insulin secretion and clearance. To measure insulin secretion, we assessed the plasma concentration of C-peptide during ipGTT. After glucose administration, the HFD group presented increased C-peptide levels during the test (Fig. 2C), indicating higher insulin secretion in this group compared to CON mice (Fig. 2D). Also, plasma insulin concentration was increased in the HFD mice (Fig. 2A and B), reducing the C-peptide:insulin ratio. Insulin and C-peptide are co-secreted by the pancreatic β cells (ratio 1:1); however C-peptide has a longer half-time than insulin. Thus, reduction in the C-peptide:insulin ratio indicates a reduced insulin clearance, as we observed in the HFD mice (Fig. 2E and F). The TUDCA treatment, in HFD mice, did not alter the higher insulin secretion, as we observed by the elevated plasma C-peptide concentration during the ipGTT (Fig. 2C and D); however, it reduced plasma insulin concentration (Fig. 2A and B), restoring the C-peptide:insulin ratio to similar levels of CON group (Fig. 2E and F), indicating a reestablishment of insulin clearance in these HFD + TUDCA mice. To elucidate the mechanism whereby TUDCA restored insulin clearance in HFD mice, we also investigated the IDE expression and activity in the liver of these mice. As expected, IDE expression (Fig. 3A) and activity (Fig. 3B and C) are reduced in HFD mice, supporting the lower insulin clearance in this group. Although TUDCA treatment did not alter IDE activity (Fig. 3B and C), it restored the IDE protein expression in the HFD + TUDCA to levels similar to that of CON mice (Fig. 3A), explaining at least in part the reestablishment of insulin clearance in these mice.

### TUDCA increased IDE expression in HepG2 cells by an S1PR2–IR pathway dependent mechanism

**Figure 4:**
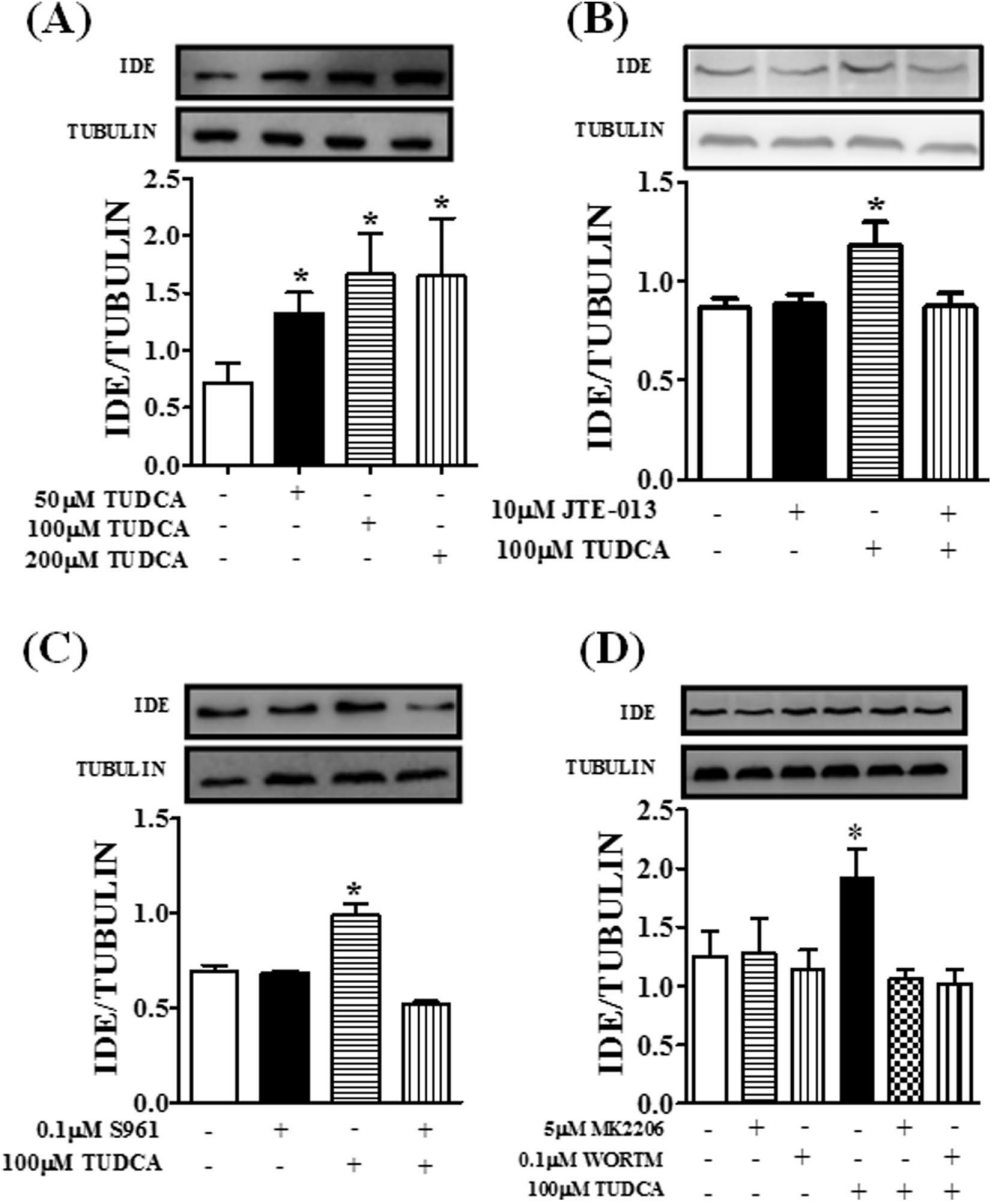
TUDCA modulates IDE expression in HepG2 cells by a S1PR2 -IR receptor pathway. Protein expression of IDE in HepG2 cells treated or not with different concentrations of TUDCA for 24-h (**A**). Effect of TUDCA on IDE expression in the presence of 10 µM sphingosine-1-phosphate receptor 2 inhibitor (JTE-013) (**B**), 0.1 µM insulin receptor inhibitor (S961) (**C**) and 5 µM of Akt inhibitor (MK2206) or 0.1 µM of PI3k inhibitor (Wortmannin) (**D**). Data are mean ± SEM (n = 4–6). *P ≤ 0.05 *vs* control conditions.

To assess the direct effect of TUDCA on IDE expression, we performed *in vitro* experiments using human liver cell line HepG2. First, these cells were exposed to different concentrations of TUDCA; after 24 h incubation, we observed that TUDCA increased IDE expression at 50, 100 and 200 µM (Fig. 4A). Thus, in the subsequent experiments, we used 100 µM TUDCA. It is known that the activation of the insulin pathway increases IDE expression in neurons^32^ and the bile acid TUDCA activates the insulin pathway mainly by the S1PR2 receptor in the liver^30^. Figure 4B shows that in the presence of the S1PR2 inhibitor, JTE-013, TUDCA failed to increase the expression of IDE. In addition, TUDCA also failed to increase IDE expression in the cells incubated with S196 (an IR inhibitor)^33^, MK-2206 (an Akt inhibitor) or Wortmannin (a PI3K inhibitor)^34^ (Fig. 4C and D, respectively).

## Discussion

Plasma insulin concentration is controlled by the interaction between insulin secretion and insulin clearance^7,8^. Prolonged hyperinsulinemia is associated with reduced IR tyrosine kinase activity, and the normalization of plasma insulin concentrations recovers insulin signaling^3^. In addition, non-obese mice, with over-expression of the insulin gene, present high insulin levels associated with impaired insulin sensitivity and, consequently, T2D^35^. Therefore, therapeutic interventions targeting insulin clearance could be relevant to treatment and/or prevention of this pathology. Here, we demonstrate that 15 days of TUDCA treatment ameliorates insulin clearance in HFD mice, probably by increasing IDE expression in the liver. In addition, we also demonstrate that TUDCA increases IDE expression in HepG2 cells, through a mechanism dependent on activation of the S1PR2/Insulin pathway.

As already shown^31^, we observed that TUDCA treatment decreased body weight due to a reduction in fat pad deposits, which was associated with improved glucose tolerance and insulin sensitivity. Similarly, 30 days of treatment with TUDCA also improved insulin signaling in the liver of ob/ob mice^29^. This improvement could be explained at least in part by the reduction of ER stress induced by TUDCA, which acts as a potent chemical chaperone enhancing ER capacity and protein folding activity. The improvement of ER stress induced by TUDCA also occurs in the hypothalamus, which in turn, decreased the body weight of high fat diet fed mice, by increased their energy expenditure through a stimulation of UCP1 protein expression in the white and brown adipose tissue, without alterations on food intake^36^.

In both the HFD and HFD + TUDCA groups, plasma C-peptide concentrations were increased. In HFD mice, this increase indirectly reflects augmented insulin secretion, probably as a consequence of insulin resistance^37^. In the HFD + TUDCA mice, which had lower insulin resistance than HFD mice, the increased insulin secretion may be a direct effect of TUDCA on pancreatic islets^38^.

The mechanism whereby TUDCA increases insulin secretion has been investigated. In pancreatic islets of pigs, TUDCA reduced ER stress induced by thapsigargin and increased insulin secretion, suggesting this bile acid as an important enhancer of islet function^39^. Furthermore, in pancreatic islets of mice incubated with a cAMP competitor or a PKA inhibitor, the increase of insulin secretion induced by TUDCA was blunted, indicating the involvement of this pathway in the mechanism whereby TUDCA increases the secretion of this hormone^38^.

Although the effect of TUDCA upon insulin clearance had not been investigated before, previous data indicated a possible role of this bile acid in the degradation of this hormone. It was observed that 30 days of treatment with TUDCA reduced plasma insulin concentration in ob/ob mice^29^. Considering that TUDCA increased insulin secretion in pancreatic islets^38,39^, the lower insulinemia found in those obese mice could possibly be explained by changes in insulin clearance. Indeed, we demonstrated that treatment with TUDCA increases insulin removal in HFD mice, suggesting this bile acid as an important insulin clearance booster. We believe that this effect of TUDCA could be due to increased protein expression of IDE, the main enzyme responsible for insulin degradation^7^, since the activity of this enzyme did not change in the liver of HFD + TUDCA mice.

In an attempt to explore the direct effect of TUDCA upon IDE expression, we incubated the human hepatic cell line HepG2, with or without TUDCA, and we confirmed that TUDCA *per se* increases IDE expression in these cells. These data reinforce our premise that this bile acid may improve insulin clearance by increasing IDE expression in the liver, contributing to the normalization of plasma insulin levels in hyperinsulinemic pre-diabetic mice.

In addition to the well-known effects of bile acids as regulators of lipid digestion and absorption in the small intestine^40^, it was suggested that they also act as hormones, dependent on the bile acid type binding with specific receptors^38–42^. In the hepatic cell TUDCA is a ligand of a G-protein coupled protein receptor called S1PR2^30^. The binding of TUDCA and other conjugated bile acids to S1PR2 activates the insulin pathway at the IR-PI3K-Akt level. Here, we show that TUDCA increases IDE expression in hepatic cells and this effect seems to be dependent on S1PR2-IR-PI3K-Akt activation pathway.

Previous studies have demonstrated that different interventions that improve insulin signaling are associated with increased insulin clearance and IDE expression in obese mice^19,20^. In fact, neural cells exposed to insulin showed increased IDE expression, whereas insulin pathway inhibition, at the PI3K level, avoided the insulin-induced IDE expression^32^. This evidence supports our findings about the possible role of the insulin pathway over IDE expression.

Although this evidence indicates that increased insulin-signaling might induce IDE expression, others studies have interpreted the evidence differently, suggesting that IDE expression could also affect the insulin pathway^43^. Human subjects, with a polymorphism at the *IDE* gene, develop insulin resistance and T2D^44^. Likewise, Goto-Kakizaki rats, which have a defect at the *IDE* gene, develop hyperinsulinemia, insulin resistance and, ultimately, T2D^17^. Also, IDE knockout mice are hyperinsulinemic, which contributes to chronic insulin signaling stimulation, which in turn results in insulin resistance through reduced IR expression in skeletal muscle, adipose tissue and the liver^16^.

Our data confirm the important role of TUDCA in the regulation of insulin signaling and secretion, and further elucidates the function of this bile acid on insulin metabolism and its importance in pathological conditions such as obesity and T2D. Although elevated serum bile acids were observed in T2D patients^45^, improved glucose metabolism in obese patients was related to the increase of serum bile acids after bariatric surgery^46^. This phenomenon seems to be due to the higher expression of enzymes responsible for bile acid synthesis and conjugation, such as cholesterol 7α hydroxylase (CYP7A1), bile acid: CoA synthase (BACS) and bile acid-CoA: amino acid N-acyltransferase (BAAT)^47^. Indeed, overexpression of CYP7A1, which increased bile acid synthesis, prevented high fat diet-induced obesity and also insulin resistance in mice^48^, pointing bile acids as an interesting target to fight these pathologies.

In conclusion, our outcomes provide evidence that TUDCA may be a therapeutic strategy to counteract obesity-induced hyperinsulinemia. We found that TUDCA increased insulin clearance in HFD mice, probably through increased IDE expression in the liver, reestablishing their plasma insulin levels. Our results also indicated that TUDCA-induced IDE expression seems to be mediated by the S1PR2-Insulin signaling pathway.

## Materials and Methods

### Reagents

TUDCA was purchased from Calbiochem (Sao Paulo, Brazil, cat. 580549) and Insulin and C-Peptide Elisa Kits were acquired from Millipore (Darmstadt, Germany, cat. #EZRMI-13K and #EZRMCP2-21K, respectively). Western Blot reagents were purchased from Bio-Rad (Madrid, Spain) and antibodies were acquired from Abcam (Cambridge, UK) and Sigma Aldrich (St Louis, MO, USA). The remaining reagents were purchased from Sigma Aldrich.

### Animals

The experiments involving animals were approved by the Animal Care Committee at UNICAMP (license number: 3815-1) and were conducted in accordance to the last revision of the National Institutes of Health (NIH) guide for the care and use of laboratory animal. Male 21-days old C57Bl/6 mice were obtained from the breeding colony at UNICAMP and maintained at 22 ± 1 °C, on a 12-h light–dark cycle. After 1 month, the mice were fed a standard diet (CON) or a high fat diet with 35% fat (HFD) during 12 weeks. On the last 15 days of this period, the mice received, i.p., PBS (groups CON and HFD) or 300 mg/kg TUDCA (groups CON + TUDCA and HFD + TUDCA). The mice were killed in a CO_2_ chamber and decapitated for blood collection and removal of the liver for posterior analyses.

### Intraperitoneal Glucose and Insulin Tolerance Tests

At the end of treatment with TUDCA, the mice were subjected to 12-h fasting to perform the ipGTT. The fasting blood glucose level were measured (time 0) by a glucometer. After, the mice received an i.p. glucose load of 2 g/kg body weight and the glycemia was measured at 15, 30, 60 and 120 min after the glucose load. For the ipITT, the mice were subjected to a 2-h fasting and the glycemia was measured before (time 0) and 3, 6, 9, 12, 15 and 18 min after the i.p. administration of 0.75 U/kg insulin load. The KITT was calculated as previously described^49^.

### Plasma Insulin and C-peptide measurements

Mouse insulin and C-peptide Elisa Kits (Darmstadt, Germany, cat. #EZRMI-13K and #EZRMCP2-21K, respectively) were used to measure plasma insulin and C-peptide. The plasma samples were obtained by centrifugation of blood samples at 1100 g, 15 min, 4 °C. The assays were performed as indicated on kit protocol. The blood samples for insulin measurements were collected on fed and fasted state, as well as at the ipGTT times 0, 15 and 60 min. The C-peptide was measured in these same plasma samples of ipGTT, used for insulin measurements.

### Cell culture and treatment

HepG2 liver cell line were cultured in DMEN (Vitrocell, Campinas, SP, Brazil), enriched with 10% (vol./vol.) fetal bovine serum (FBS) for 3 days, under a humidified condition with 5% CO_2_ at 37 °C. After that, the cells were incubated in the presence, or not, of different TUDCA concentrations (T50, T100 and T200 µM) during 24-h. The concentration of 100 µM was adopted for the following experiments. The inhibitors of insulin pathway and bile acid receptors were added when necessary, as describe on figure legends. In the experiments with S961, MK2206 or Wortmannin, the cells were submitted to a 6-h serum starved before treatment.

### Western blot analysis

Liver samples were collected and homogenized with 500 µL of Cell Lysis Buffer. For HepG2 Western blot, after treatment, the cells were collected in trypsin/EDTA, washed with PBS, and homogenized in urea anti-protease/anti-phosphatase buffer. For SDS (sodium dodecyl sulfate) polyacrylamide gel electrophoresis, all samples were treated with a Laemmli buffer containing dithiothreitol. After heating to 95 °C for 5 min, proteins were separated by electrophoresis in a 10% polyacrylamide gel. The transfer to nitrocellulose membranes was performed in a Trans Blot transfer for 2-h in 100 V, with Tris/Glycine buffer. After, the membranes were blocked with 5% non-fat dry milk buffer (5% milk, 10 mM TRIS, 150 mM NaCl and Tween 20 0.02%) during 1-h, and then, they were incubated with a polyclonal antibody against IDE (Abcam, cat. ab32216). Tubulin (Sigma Aldrich, cat. 6074) was used as control of the experiment. Visualization of specific protein bands was performed by incubating the membranes with appropriate secondary antibodies. Protein bands were visualized using the Amersham Imager 600 (GE Healthcare Life Sciences, Buckinghamshire, UK) which detects the chemiluminescence. The band intensities were quantified with the Image J software (National Institutes of Health, Bethesda, MD, USA).

### IDE activity

Liver IDE activity measurement was performed using the SensoLyte 520 IDE Activity Assay Kit (AnaSpec, Fremont, CA, USA, cat. AS-72231) following the manufacturer’s instructions. The total IDE activity was calculated as previously described^19^ and normalized per μg of total protein, which was determined using Bradford reagent. The kinetic concentration of 5-FAM was also normalized per μg of total protein.

### Statistical analysis

The data were presented as means ± standard errors media (SEM) for 4–8 animals, or obtained for 3 different cells experiments, each one in triplicate. The comparisons between all groups were performed by one-way ANOVA analysis followed by Newman-Keuls test. When the comparisons were determined between two groups Student´s t-test was adopted. The difference between the groups were considered statistically significant if P ≤ 0.05.

## Electronic supplementary material

Supplementary Information

## References

[1] Hsu IR, Kim SP, Kabir M, Bergman RN (2007). Metabolic syndrome, hyperinsulinemia, and cancer. Am J Clin Nutr.

[2] Yun JE, Won S, Sung J, Jee SH (2012). Impact of metabolic syndrome independent of insulin resistance on the development of cardiovascular disease. Circ J.

[3] Kanety H, Moshe S, Shafrir E, Lunenfeld B, Karasik A (1994). Hyperinsulinemia induces a reversible impairment in insulin receptor function leading to diabetes in the sand rat model of non-insulin-dependent diabetes mellitus. Proc Natl Acad Sci USA.

[4] Weyer C, Bogardus C, Mott DM, Pratley RE (1999). The natural history of insulin secretory dysfunction and insulin resistance in the pathogenesis of type 2 diabetes mellitus. J Clin Invest.

[5] Brandimarti P (2013). Cafeteria diet inhibits insulin clearance by reduced insulin-degrading enzyme expression and mRNA splicing. J Endocrinol.

[6] Erdmann J (2009). Weight-dependent differential contribution of insulin secretion and clearance to hyperinsulinemia of obesity. Regul Pept.

[7] Duckworth WC, Kitabchi AE (1981). Insulin metabolism and degradation. Endocr Rev.

[8] Duckworth WC, Bennett RG, Hamel FG (1998). Insulin degradation: progress and potential. Endocrine reviews.

[9] Rose K (1988). Insulin proteinase liberates from glucagon a fragment known to have enhanced activity against Ca2++ Mg2+ -dependent ATPase. Biochem J.

[10] Bennett RG, Duckworth WC, Hamel FG (2000). Degradation of amylin by insulin-degrading enzyme. J Biol Chem.

[11] Farris W (2003). Insulin-degrading enzyme regulates the levels of insulin, amyloid beta-protein, and the beta-amyloid precursor protein intracellular domain *in vivo*. Proc Natl Acad Sci USA.

[12] Karamohamed S (2003). Polymorphisms in the insulin-degrading enzyme gene are associated with type 2 diabetes in men from the NHLBI Framingham Heart Study. Diabetes.

[13] Bertram L (2000). Evidence for genetic linkage of Alzheimer’s disease to chromosome 10q. Science.

[14] Maianti JP (2014). Anti-diabetic activity of insulin-degrading enzyme inhibitors mediated by multiple hormones. Nature.

[15] Deprez-Poulain R (2015). Catalytic site inhibition of insulin-degrading enzyme by a small molecule induces glucose intolerance in mice. Nat Commun.

[16] Abdul-Hay SO (2011). Deletion of insulin-degrading enzyme elicits antipodal, age-dependent effects on glucose and insulin tolerance. PLoS One.

[17] Fakhrai-Rad H (2000). Insulin-degrading enzyme identified as a candidate diabetes susceptibility gene in GK rats. Hum Mol Genet.

[18] Kotronen A, Juurinen L, Tiikkainen M, Vehkavaara S, Yki-Jarvinen H (2008). Increased liver fat, impaired insulin clearance, and hepatic and adipose tissue insulin resistance in type 2 diabetes. Gastroenterology.

[19] Kurauti MA (2016). Acute exercise restores insulin clearance in diet-induced obese mice. J Endocrinol.

[20] Bojsen-Møller KN (2013). Increased hepatic insulin clearance after Roux-en-Y gastric bypass. J Clin Endocrinol Metab.

[21] Wei X (2014). Regulation of insulin degrading enzyme activity by obesity-associated factors and pioglitazone in liver of diet-induced obese mice. PLoS One.

[22] Dalle Grave R, Calugi S, Centis E, El Ghoch M, Marchesini G (2011). Cognitive-behavioral strategies to increase the adherence to exercise in the management of obesity. J Obes.

[23] Carlsson LM (2012). Bariatric surgery and prevention of type 2 diabetes in Swedish obese subjects. N Engl J Med.

[24] Shah P, Mudaliar S (2010). Pioglitazone: side effect and safety profile. Expert Opin Drug Saf.

[25] da-Silva WS (2011). The chemical chaperones tauroursodeoxycholic and 4-phenylbutyric acid accelerate thyroid hormone activation and energy expenditure. FEBS Lett.

[26] Turdi S, Hu N, Ren J (2013). Tauroursodeoxycholic acid mitigates high fat diet-induced cardiomyocyte contractile and intracellular Ca2+ anomalies. PLoS One.

[27] Yang JS (2010). Changes in hepatic gene expression upon oral administration of taurine-conjugated ursodeoxycholic acid in ob/ob mice. PLoS One.

[28] Xie Q (2002). Effect of tauroursodeoxycholic acid on endoplasmic reticulum stress-induced caspase-12 activation. Hepatology.

[29] Ozcan U (2006). Chemical chaperones reduce ER stress and restore glucose homeostasis in a mouse model of type 2 diabetes. Science.

[30] Studer E (2012). Conjugated bile acids activate the sphingosine-1-phosphate receptor 2 in primary rodent hepatocytes. Hepatology.

[31] Guo Q (2015). Glycolipid Metabolism Disorder in the Liver of Obese Mice Is Improved by TUDCA via the Restoration of Defective Hepatic Autophagy. Int J Endocrinol.

[32] Zhao L (2004). Insulin-degrading enzyme as a downstream target of insulin receptor signaling cascade: implications for Alzheimer’s disease intervention. J Neurosci.

[33] Vikram A, Jena G (2010). S961, an insulin receptor antagonist causes hyperinsulinemia, insulin-resistance and depletion of energy stores in rats. Biochem Biophys Res Commun.

[34] Pal SK, Reckamp K, Yu H, Figlin RA (2010). Akt inhibitors in clinical development for the treatment of cancer. Expert Opin Investig Drugs.

[35] Marban SL, DeLoia JA, Gearhart JD (1989). Hyperinsulinemia in transgenic mice carrying multiple copies of the human insulin gene. Dev Genet.

[36] Contreras C (2017). Reduction of Hypothalamic Endoplasmic Reticulum Stress Activates Browning of White Fat and Ameliorates Obesity. Diabetes.

[37] Araújo TG, Oliveira AG, Saad MJ (2013). Insulin-resistance-associated compensatory mechanisms of pancreatic Beta cells: a current opinion. Front Endocrinol (Lausanne).

[38] Vettorazzi JF (2016). The bile acid TUDCA increases glucose-induced insulin secretion via the cAMP/PKA pathway in pancreatic beta cells. Metabolism.

[39] Lee YY (2010). Tauroursodeoxycholate (TUDCA), chemical chaperone, enhances function of islets by reducing ER stress. Biochem Biophys Res Commun.

[40] Chiang JY (2013). Bile acid metabolism and signaling. Compr Physiol.

[41] Cipriani S (2011). The bile acid receptor GPBAR-1 (TGR5) modulates integrity of intestinal barrier and immune response to experimental colitis. PLoS One.

[42] Fiorucci S, Mencarelli A, Palladino G, Cipriani S (2009). Bile-acid-activated receptors: targeting TGR5 and farnesoid-X-receptor in lipid and glucose disorders. Trends Pharmacol Sci.

[43] Galagovsky D (2014). The Drosophila insulin-degrading enzyme restricts growth by modulating the PI3K pathway in a cell-autonomous manner. Mol Biol Cell.

[44] Pivovarova O (2013). Hepatic insulin clearance is closely related to metabolic syndrome components. Diabetes Care.

[45] Wewalka M, Patti ME, Barbato C, Houten SM, Goldfine AB (2014). Fasting serum taurine-conjugated bile acids are elevated in type 2 diabetes and do not change with intensification of insulin. J Clin Endocrinol Metab.

[46] Patti ME (2009). Serum bile acids are higher in humans with prior gastric bypass: potential contribution to improved glucose and lipid metabolism. Obesity (Silver Spring).

[47] Wu Q (2016). Effects of Bariatric Surgery on Serum Bile Acid Composition and Conjugation in a Diabetic Rat Model. Obes Surg.

[48] Li T (2010). Transgenic expression of cholesterol 7alpha-hydroxylase in the liver prevents high-fat diet-induced obesity and insulin resistance in mice. Hepatology.

[49] Akinmokun A, Selby PL, Ramaiya K, Alberti KG (1992). The short insulin tolerance test for determination of insulin sensitivity: a comparison with the euglycaemic clamp. Diabet Med.

