# Supplementary material for: Bile acid TUDCA improves insulin clearance by increasing the expression of insulin-degrading enzyme in the liver of obese mice
Source: Sci Rep. 2017 Nov 1;7:14876. doi: 10.1038/s41598-017-13974-0 (PMC5665899; doi:10.1038/s41598-017-13974-0)

Bile acid TUDCA improves insulin clearance by increasing the expression of insulin-degrading enzyme in the liver of obese mice

Jean Franciesco Vettorazzi[a], Mirian Ayumi Kurauti[a], Gabriela Moreira Soares, Patricia Cristine Borck, Sandra Mara Ferreira, Renato Chaves Souto Branco, Luciana de Souza Lima Michelone, Antonio Carlos Boschero, Jose Maria Costa Junior* & Everardo Magalhães Carneiro*#

Department of Structural and Functional Biology, Institute of Biology, University of Campinas (UNICAMP), 13083-970 Campinas, SP, Brazil

a These authors contributed equally to this study

* These authors contributed equally to the conception and supervision of this study

Full unedited gel from Figure 03
Groups: CON, CON + TUDCA, HFD, HFD + TUDCA (Membrane 01 and 02)
CON, HFD, HF + TUDCA (Membrane 03)

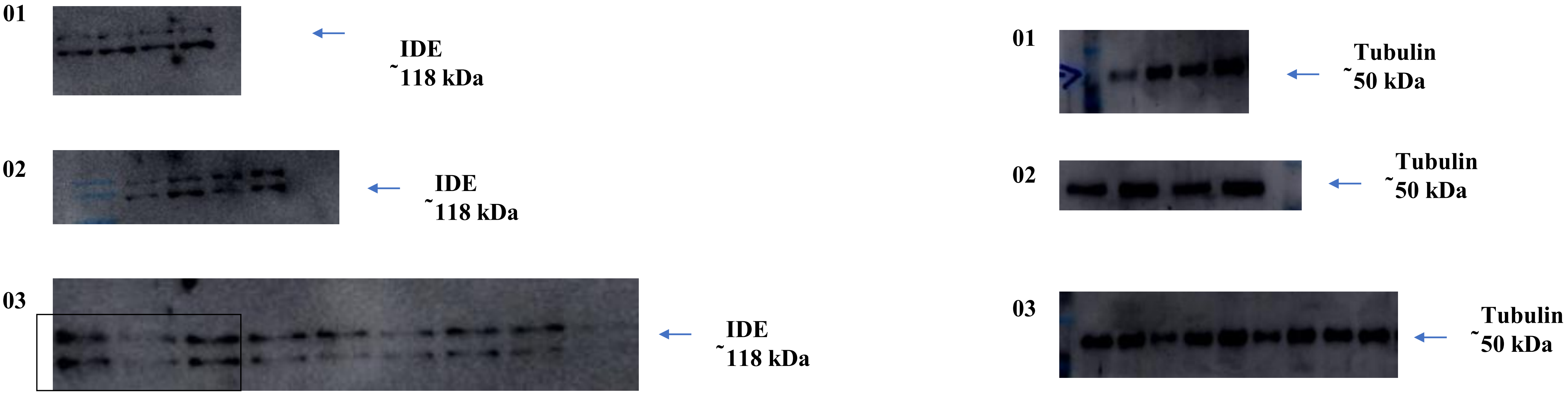

Full unedited gel from Figure 04A
Groups: CON, T50, T100, T200

01

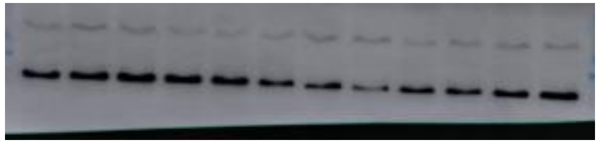

IDE ~118 kDa

02

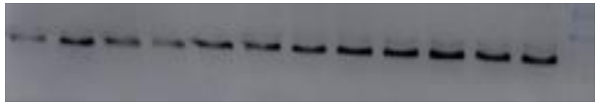

IDE ~118 kDa

03

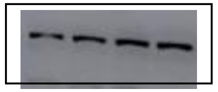

IDE ~118 kDa

01

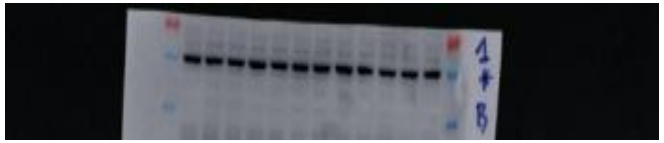

Tubulin ~50 kDa

02

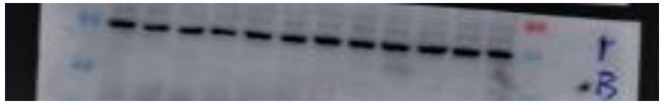

Tubulin ~50 kDa

03

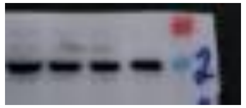

Tubulin ~50 kDa

Full unedited gel from Figure 04B

Groups: CON, CON + JTE-013, TUDCA, TUDCA + JTE-013

01

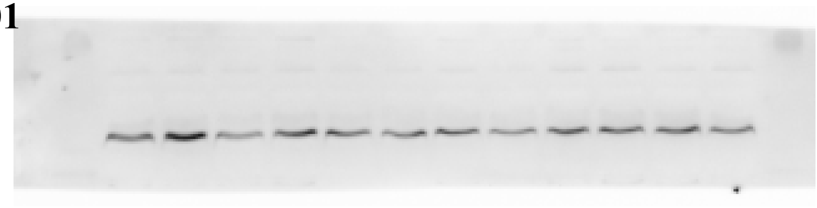

IDE ~118 kDa

02

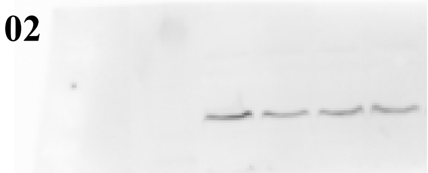

IDE ~118 kDa

03

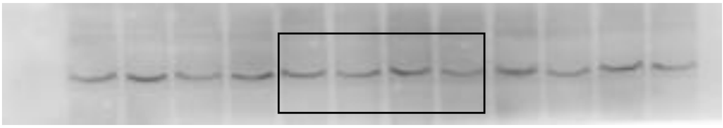

IDE ~118 kDa

04

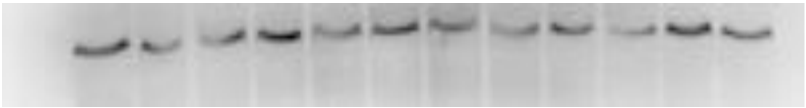

IDE ~118 kDa

01

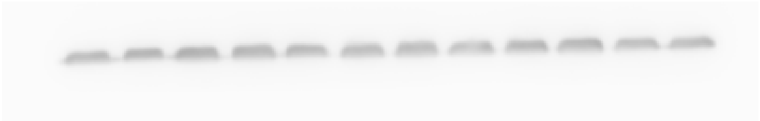

Tubulin ~50 kDa

02

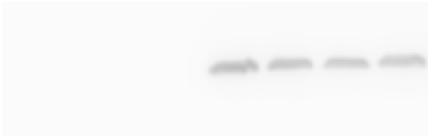

Tubulin ~50 kDa

03

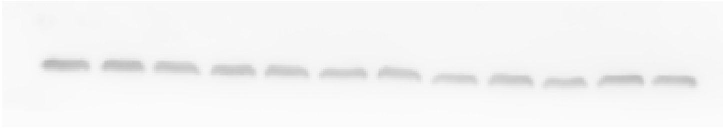

Tubulin ~50 kDa

04

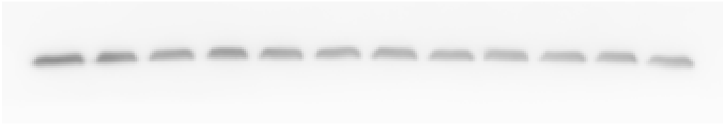

Tubulin ~50 kDa

Full unedited gel from Figure 04C
Groups: CON, CON + S961, TUDCA, TUDCA + S961

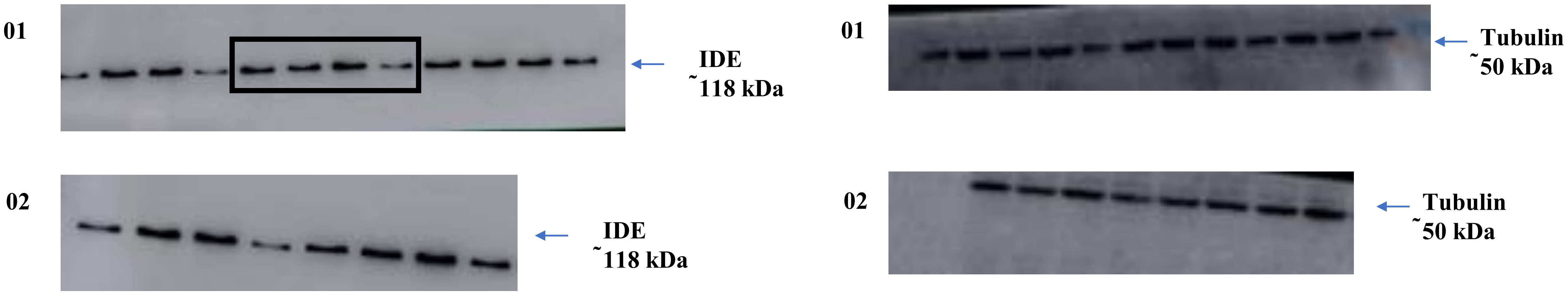

Full unedited gel from Figure 04D

Groups: CON, CON + MK2206, CON + Wortmannin, TUDCA, TUDCA + MK2206, TUDCA + Wortmannin

Full unedited gel from Figure 04D

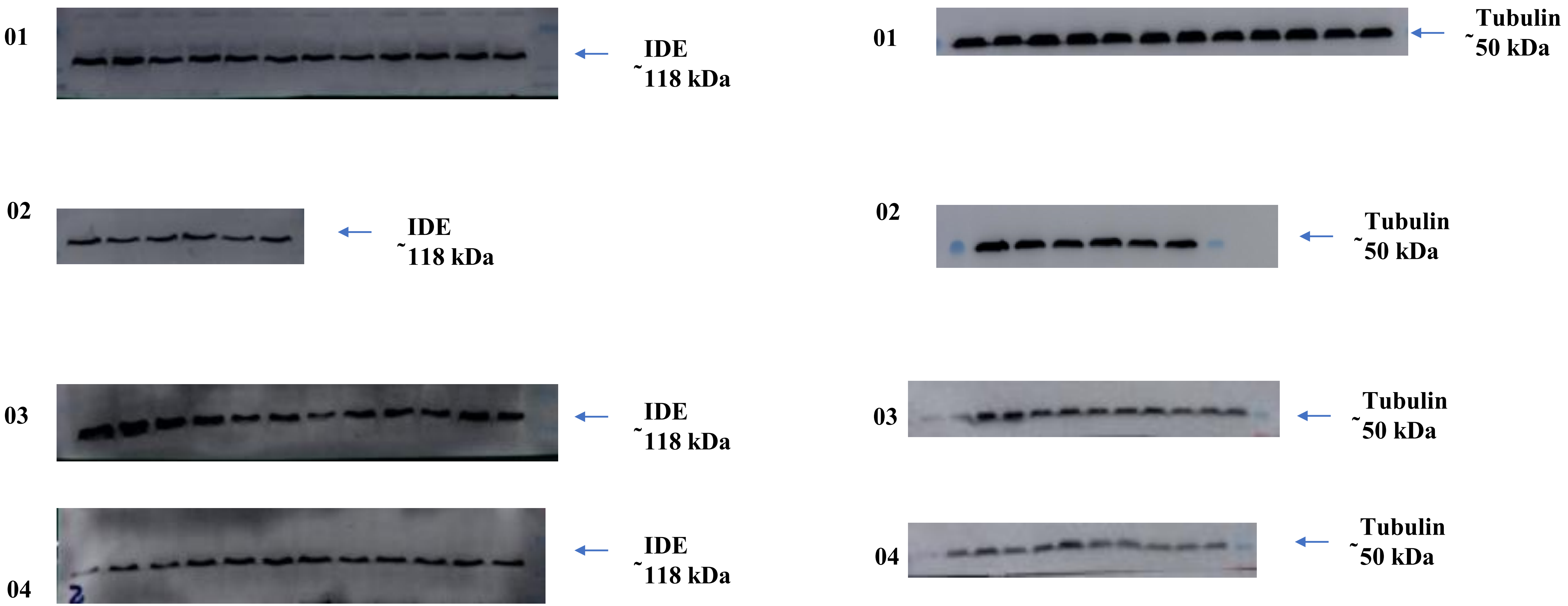

Supplement: Supplementary file 1 — Supplementary Information [file 41598_2017_13974_MOESM1_ESM.pdf]
